# A Conserved Structural Role for the Walker-A Lysine in P-Loop Containing Kinases

**DOI:** 10.3389/fmolb.2021.747206

**Published:** 2021-10-01

**Authors:** Fatlum Hajredini, Ranajeet Ghose

**Affiliations:** ^1^ Department of Chemistry and Biochemistry, The City College of New York, New York, NY, United States; ^2^ PhD Program in Biochemistry, The Graduate Center of CUNY, New York, NY, United States; ^3^ PhD Program in Chemistry, The Graduate Center of CUNY, New York, NY, United States; ^4^ PhD Program in Physics, The Graduate Center of CUNY, New York, NY, United States

**Keywords:** P-loop, protein kinases, shikimate kinase, molecular dynamics (MD), bacterial tyrosine kinase

## Abstract

Bacterial tyrosine kinases (BY-kinases) and shikimate kinases (SKs) comprise two structurally divergent P-loop containing enzyme families that share similar catalytic site geometries, most notably with respect to their Walker-A, Walker-B, and DxD motifs. We had previously demonstrated that in BY-kinases, a specific interaction between the Walker-A and Walker-B motifs, driven by the conserved “catalytic” lysine housed on the former, leads to a conformation that is unable to efficiently coordinate Mg^2+^•ATP and is therefore incapable of chemistry. Here, using enhanced sampling molecular dynamics simulations, we demonstrate that structurally similar interactions between the Walker-A and Walker-B motifs, also mediated by the catalytic lysine, stabilize a state in SKs that deviates significantly from one that is necessary for the optimal coordination of Mg^2+^•ATP. This structural role of the Walker-A lysine is a general feature in SKs and is found to be present in members that encode a Walker-B sequence characteristic of the family (*Coxiella burnetii* SK), and in those that do not (*Mycobacterium tuberculosis* SK). Thus, the structural role of the Walker-A lysine in stabilizing an inactive state, distinct from its catalytic function, is conserved between two distantly related P-loop containing kinase families, the SKs and the BY-kinases. The universal conservation of this element, and of the key characteristics of its associated interaction partners within the Walker motifs of P-loop containing enzymes, suggests that this structural role of the Walker-A lysine is perhaps a widely deployed regulatory mechanism within this ancient family.

## Introduction

P-loop containing proteins are widespread in all three branches of the tree of life suggesting that they emerged prior to the last universal common ancestor (LUCA) (Aravind et al., 2002; Edwards et al., 2013; Longo et al., 2020). Nucleotide triphosphatases (NTPases) (Leipe et al., 2002) and P-loop kinases constitute two of several divergent families of P-loop containing enzymes that encode sequences known as Walker-A (GxxxGK[S/T], x is any residue) and Walker-B (ϕϕϕϕ[D/E]xxG, ϕ is a hydrophobic residue) motifs, or variations thereon (Walker et al., 1982). The BY-kinases (for Bacterial tYrosine kinases), that are widely conserved within the prokaryotic kingdom, but are without archaeal or eukaryotic counterparts (Shi et al., 2014), constitute a unique family of protein tyrosine kinases within the SIMIBI (named for the signal-recognition GTPases, MinD and BioD superfamilies) class of NTPases (Leipe et al., 2002). Instead of the dual-lobed fold characteristic of the eukaryotic protein kinases (Taylor and Radzio-Andzelm, 1994; Scheeff and Bourne, 2005), the catalytic domains (CDs) of BY-kinases (Lee and Jia, 2009; Grangeasse et al., 2012) utilize a fold that closely resembles ATPases of the MinD family. While BY-kinase CDs may be classified as P-loop kinases based on their function, they deviate significantly from the P-loop kinase superfamily (comprising kinases that phosphorylate small molecules e.g., nucleosides, shikimate, 6-phosphofructose etc.) as defined by Leipe *et al.* (Leipe et al., 2003) (we will adhere to that definition here), notably through the absence of a “LID” domain present in the latter (Supplementary Figure S1A). In addition to modified Walker-A and Walker-B motifs, BY-kinase CDs also encode a sequence, ϕϕϕϕDxDxR, that has been termed the Walker-A′ motif (Grangeasse et al., 2012). The DxD sequence present in the Walker-A’ motif is also found in many members of the SIMIBI class of P-loop enzymes including MinD (Supplementary Figure S1B). Interestingly, the DxD motif, is characteristic of many P-loop kinases including those that utilize shikimate, gluconate or adenosine 5’-phosphosulfate (APS) as substrates (Leipe et al., 2003). A key feature of these “DxD-group” P-loop kinases is a Walker-B motif that is degraded at its C-terminal end and marked by the conspicuous absence of the acidic Asp (or Glu) residue (Supplementary Figure S1C) (Leipe et al., 2003). While the BY-kinase CD and P-loop kinases deviate significantly in their three-dimensional structures, they display similar catalytic site geometries formed by closely related structural motifs (Supplementary Figure S1C). Given the close similarities in the catalytic site architecture despite being embedded in divergent structural scaffolds, and the fact that both families perform similar phospho-transfer chemistry, we wondered whether these two distantly related families retain common structural/dynamic features that characterize their functional states.

We have previously demonstrated the unique conformational dynamics of the catalytic core of the archetypal BY-kinase, *Escherichia coli* (K12) Wzc (Wzc_CDΔC_); these dynamics appear to have significant functional consequences. Our studies revealed the presence of two major conformations with distinct long- and short-range structural features (Hajredini et al., 2020) in Wzc_CDΔC_. We designated these conformations as open (open state, OS) and closed (closed state, CS) based on their global compactness. The OS has high affinity for ADP but is unable to efficiently engage ATP•Mg^2+^. The CS, that can optimally engage ATP•Mg^2+^, is stabilized upon formation of a closed octameric ring seen both in the structure of the isolated CD (Bechet et al., 2010) as well as in the membrane-anchored full-length protein (Yang et al., 2021). Oligomer formation is necessary to enable intermolecular autophosphorylation. Thus, the differential nucleotide preferences of the OS and CS together with the coupling of their relative stabilities to oligomerization appear to play a critical role in maintaining optimal Wzc function through modulation of the substrate affinity and the prevention of futile ATP hydrolysis (Hajredini et al., 2020). A key structural feature that was found to distinguish the OS from the CS involved an alteration in the mode of interaction between conserved Walker-A and Walker-B residues (Hajredini et al., 2020; Hajredini and Ghose, 2021). In the OS, the conserved Walker-A Lys (the so-called “catalytic lysine”, K540) establishes a salt bridge with the conserved Walker-B Asp (D642) generating a conformation that cannot effectively coordinate Mg^2+^ (and consequently, ATP•Mg^2+^). In the CS, the Walker-A Lys disengages from the Walker-B Asp and the latter then contacts the conserved Walker-A Thr (T541) generating a conformation required to optimally coordinate Mg^2+^. To assess whether similar conformational states stabilized by interactions between key conserved residues also populate the landscape of the P-loop kinases, we performed enhanced sampling molecular dynamics simulations (REST2) (Wang et al., 2011) using *Mycobacterium tuberculosis* SK (SK_Mtu_ from hereon) in its unliganded, and its ATP•Mg^2+^-bound states. Our results suggest that SK_Mtu_ samples distinct global conformations that, as in the case of the Wzc_CDΔC_, are distinguished by local interactions of the Walker-A and Walker-B motifs, most notably those involving the catalytic Lys (K15). This structural feature of the Walker-A Lys in SKs is also preserved despite sequence variations in the Walker-B motif (as in *Coxiella burnetii* SK, SK_Cbu_) suggesting that this particular role of this residue in parsing functional conformations may be a general feature in P-loop containing enzymes.

## Materials and Methods

### Preparation of Starting Structures

The starting conformation of the SK_Mtu_•ATP•Mg^2+^ complex was generated using the coordinates of SK_Mtu_ from the structure of the SK_Mtu_•ADP•Mg^2+^•shikimate complex (PDB: 1WE2) (Pereira et al., 2004) after removal of the bound ADP and shikimate. The Mg^2+^ ion and all solvent molecules were retained. The coordinates of ATP were derived from the structure of the SK_Mtu_•AMPPCP•shikimate complex (PDB: 1ZYU) (Gan et al., 2006) by aligning the 1WE2 and 1ZYU structures using the Cα atoms of their respective protein components and converting the bound AMPPCP to ATP through the replacement of the relevant carbon atom by oxygen. Subsequently, the protein and shikimate coordinates of the 1ZYU structure were removed to generate the SK_Mtu_•ATP•Mg^2+^ complex. For consistency, the corresponding apo simulations utilized the same starting structure after removal of ATP•Mg^2+^. For unliganded SK_Cbu_, the starting structure was obtained directly from the coordinates found in PDB: 3TRF (Franklin et al., 2015) after removal of all ligands except solvent.

### Molecular Dynamics Simulations

The starting structures, generated as discussed above, were equilibrated using previously described protocols (Hajredini et al., 2020). Briefly, the systems were parameterized within the CHARMM36m force-field (Huang et al., 2017), solvated with TIP3P water molecules, energy minimized and then equilibrated, first in an NVT ensemble, and then in an NPT ensemble to generate starting structures for the enhanced sampling, REST2, (Wang et al., 2011) simulations. Subsequently, REST2 production runs were carried out for 200 ns using 14 replicas spanning a 300–400 K temperature range. The final exchange probabilities for the SK_Mtu_•ATP•Mg^2+^, SK_Mtu_, and SK_Cbu_ simulations were 23.3 ± 8.2, 24.2 ± 8.4, and 29.0 ± 0.6%, respectively. The first 40 ns in each of the simulations were used as “burn-in” periods and discarded, and all subsequent analyses were carried out utilizing the remaining 160 ns. As shown in Supplementary Figure S2, 40 ns was more than sufficient to ensure equilibration of the key observables (θ and |*h*|, see below) probed in the simulations.

### Definition of the Cylindrical Coordinate Frame

**FIGURE 1:**
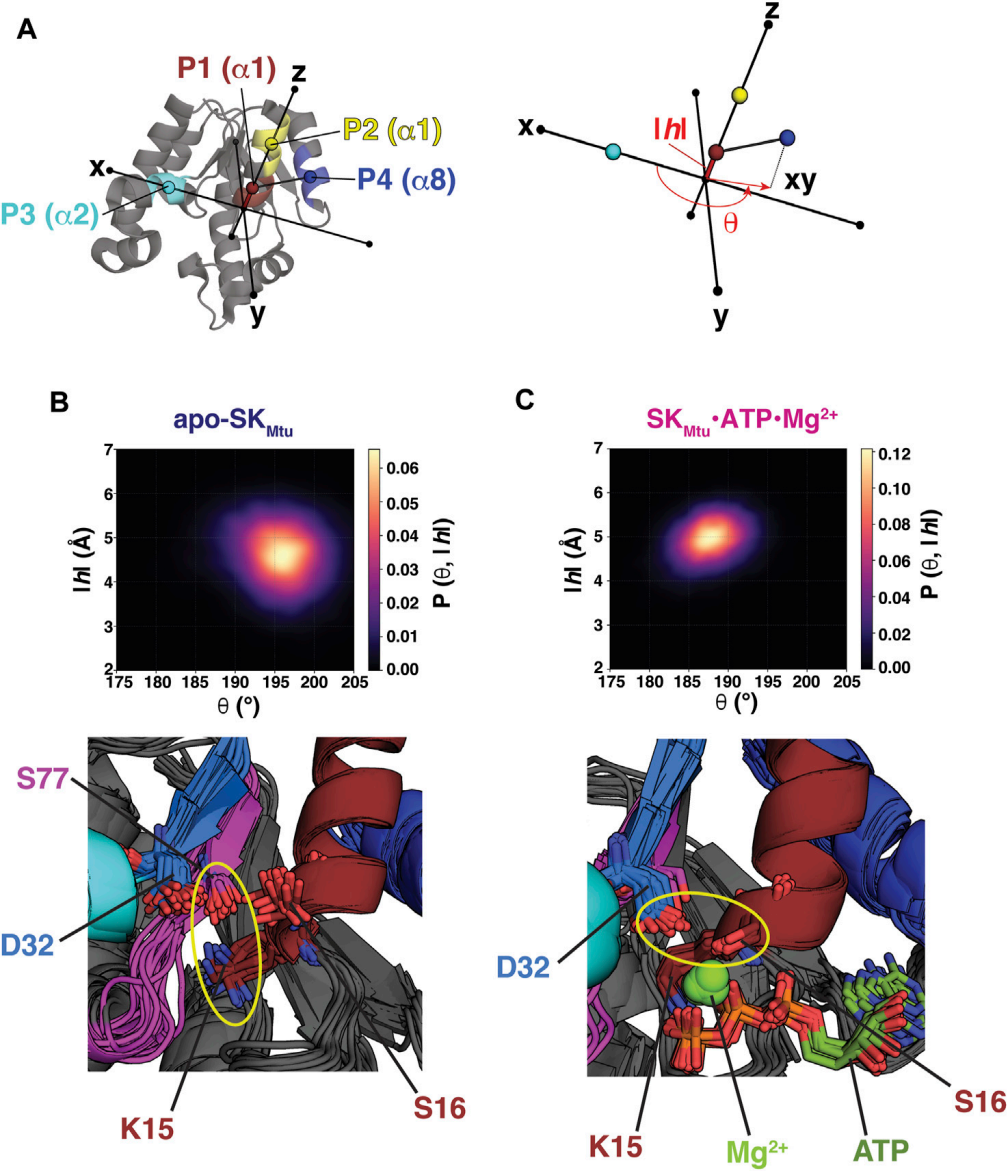
**(A)** Points P1 (brown), P2 (yellow), P3 (cyan) and P4 (dark blue) used to define the cylindrical coordinate frame for analyses of the REST2-generated ensembles of SK_Mtu_ are indicated on its structure. The definitions of the angle θ and the rise |*h*| are shown on the right panel. The REST2-generated structural ensembles of unliganded SK_Mtu_ **(B)** and the SK_Mtu_•ATP•Mg^2+^ complex **(C)** are shown projected onto θ-|*h*| space and plotted using kernel density estimation. The bottom panels show key interactions that stabilize each state using 20 representative structures drawn from the corresponding regions of highest probability density (194–200° and 4–5 Å in the |*h*| and θ dimensions, respectively for apo-SK_Mtu_ and 185–190° and 4.5–5.5 Å for the corresponding dimensions of the SK_Mtu_•ATP•Mg^2+^ complex). A key feature that distinguishes the Walker Open State (WOS) from the Walker Closed State (WCS) is the orientation of the Walker-A Lys (K15 in SK_Mtu_). For the WOS seen for apo-SK_Mtu_ K15 hydrogen bonds with S77 of the Walker-B motif in the bulk of the structures (K15 and S77 are proximal in all of them). This interaction is broken in the SK_Mtu_•ATP•Mg^2+^ complex. The yellow ellipses highlight the altered interactions of D32 in the two states.

To describe the global conformational landscape of SK_Mtu_, a cylindrical coordinate frame, comprising of an angle θ and a rise *|h|* (Figure 1A), was defined as in the case of Wzc_CDΔC_, described previously (Supplementary Figure S3A) (Hajredini et al., 2020). The centers of mass of the Cα atoms of two segments on helix α1 (that contains the Walker-A motif), comprising residues 15–19 (P1, brown) and 21–25 (P2, yellow), were used to define a vector that was aligned to the *z*-axis of the molecular frame. Each structure within the REST2-generated ensembles was rotated and translated such that point P3 (cyan), defined by the center of mass of the Cα atoms of the first turn of helix α2 (33–37), was placed on the x-axis along the positive direction. θ is then defined as the angle between the xy-projection of a vector extending from point P1 to P4 (blue; defined by the center of mass of the Cα atoms of residues 155–159 that form the first turn of helix α8). The rise *|h|* is defined as the absolute value of the distance between points P1 and P3 along the *z*-axis. Thus, the angle θ and the rise |*h*| provide measures of the outward rotation and the up/down movement, respectively, of the Walker-A motif relative to the Walker-B and DxD motifs. The crystal structures of SK_Mtu_ (listed in Supplementary Table S1) were projected onto the cylindrical coordinate frame using the same protocol. For the structures of the SK enzymes from other organisms (listed in Supplementary Table S1), a similar procedure was utilized to define the points P1-P4 (described in Supplementary Table S2) and subsequently, θ and |*h*|.

## Results and Discussion

### Unliganded and Liganded Conformational Ensembles of SK_Mtu_ Populate Distinct Structural States

We performed two sets of enhanced sampling simulations utilizing the replica exchange with solute scaling (REST2) approach. REST2 represents a modification of the Hamiltonian scaling protocol of the replica exchange with solute tempering (REST) scheme (Liu et al., 2005) that greatly enhances its efficiency by decoupling the acceptance rate from the solvent. For the REST2 simulations, protocols similar to those described before (Hajredini et al., 2020) were utilized with either unliganded (apo) or ATP•Mg^2+^-bound SK_Mtu_. To analyze the global conformational states populated by these two ensembles we utilized the cylindrical coordinate frame defined in Figure 1A. Note that this frame is similar, but not identical, to that previously defined for Wzc_CDΔC_ (Hajredini et al., 2020) (compare Supplementary Figure S3A) given the significant structural differences between the two proteins. The orientations of the α8 helix (carrying P4) and α1 (carrying P1 and P2) in SK_Mtu_ are similar to the α2-α3 pair in Wzc_CDΔC_ (the latter carries the Walker-A sequence) (Hajredini et al., 2020). *In lieu* of a missing Wzc_CDΔC_ α4 equivalent in SK_Mtu_, the α2 helix (that hosts the second Asp, D34, of the DxD motif) was utilized to define P3. Thus, while this definition allows for a qualitative comparison of the global conformational states sampled by SK_Mtu_ and Wzc_CDΔC_ with respect to their key catalytic elements, a quantitative equivalence should not be expected.

Projection of the conformational ensembles of apo-SK_Mtu_ (Figure 1B, top panel) and the SK_Mtu_•ATP•Mg^2+^ complex (Figure 1C, top panel) onto the 2-dimensional θ-|*h*| space suggests differences in global conformations between the two cases. The presence of ATP•Mg^2+^ induces a more closed conformation, characterized by smaller θ values, compared to a more open conformation (larger θ values) seen for apo-SK_Mtu_. We designate these global states as a Walker Closed State (WCS, seen in the SK_Mtu_•ATP•Mg^2+^ complex), and a Walker Open State (WOS, seen in apo-SK_Mtu_) to distinguish them from the closed and open classifications of the relative conformation of the SB and the LID domains traditionally used in the SK literature (Hartmann et al., 2006). Contrasts between the θ-|*h*| projections of SK_Mtu_ and Wzc_CDΔC_ are evident upon comparing Figures 1B,C with Supplementary Figures S3B,C. In the case of Wzc_CDΔC_, the OS is characterized by high values of both θ and |*h*|, while the CS exhibits low values for both variables (Supplementary Figures S3B,C), suggesting that these variables are positively correlated (Hajredini et al., 2020). In contrast, the regions of maximal density for the WOS (Figure 1B, top panel) and the WCS (Figure 1C, top panel) suggest a weak anti-correlation between the θ (WOS: 196 ± 4°, WCS: 188 ± 3°) and |*h*| dimensions (WOS: 4.6 ± 0.7 Å, WCS: 4.9 ± 0.4 Å), with lower θ and higher |*h*| for the WCS, and the opposite for the WOS. Further, the apparent separation between the WOS and the WCS seems less prominent than that between the OS and CS of Wzc_CDΔC_ (compare Figures 1B,C with Supplementary Figures S3B,C; also see Supplementary Figure S4A). As an independent validation of our simulations, we note that the regions in θ-|*h*| space spanned by the WOS and the WCS in our REST2 ensembles are well represented (Supplementary Figure S4A) in the several available crystal structures of SKs (and their complexes) from a variety of organisms (listed in Supplementary Table S1).

Representative structures of apo- (Figure 1B, bottom panel) and ATP•Mg^2+^-bound (Figure 1C, bottom panel) SK_Mtu_ extracted from the regions of maximal density in θ−|*h*| space of the corresponding ensembles show altered conformations of key catalytic site elements. In the presence of ATP•Mg^2+^ i.e., in the WCS, the Walker-A Lys (K15) assumes a downwards orientation and contacts the β− and γ−phosphates of ATP; the first Asp (D32) of the DxD motif forms a hydrogen bond with the Walker-A S16, thus functionally mimicking the absent Walker-B Asp (Leipe et al., 2003). The D32-S16 interaction is necessary for the optimal coordination of Mg^2+^ (Figure 1C, bottom panel). Indeed, as shown in Supplementary Figure S4B, the D32,Cγ−S16,Oγ distance is well defined and shows a narrow distribution (3.7 ± 0.2 Å) within the ensemble. In the absence of ligands, i.e., in the WOS, K15 rotates outwards to establish a hydrogen-bond with S77 of the Walker-B motif (Figure 1B, bottom panel) in a significant fraction of the structures. In this configuration, the D32-S16 distance shows a broad distribution (6.0 ± 1.2 Å), suggestive of increased disorder, with a maximum reflective of significantly increased separation (Supplementary Figure S4B) suggesting a conformation that is not conducive for Mg^2+^ coordination. Conformations displaying the K15-S77 hydrogen bond have been observed in several crystal structures of SK_Mtu_, that are, not surprisingly, free of Mg^2+^. The structures (representative examples shown in Supplementary Figure S4C) (Hartmann et al., 2006) that display the K15-S77 interaction have the highest values of θ, and correspondingly, the lowest values of |*h*|, and exist at the outer edges of the WOS. Taken together, these results suggest that the global conformations of SK_Mtu_ are coupled to the conformational state of the Walker-A Lys as was observed for Wzc_CDΔC_ (Hajredini et al., 2020; Hajredini and Ghose, 2021).

**FIGURE 2:**
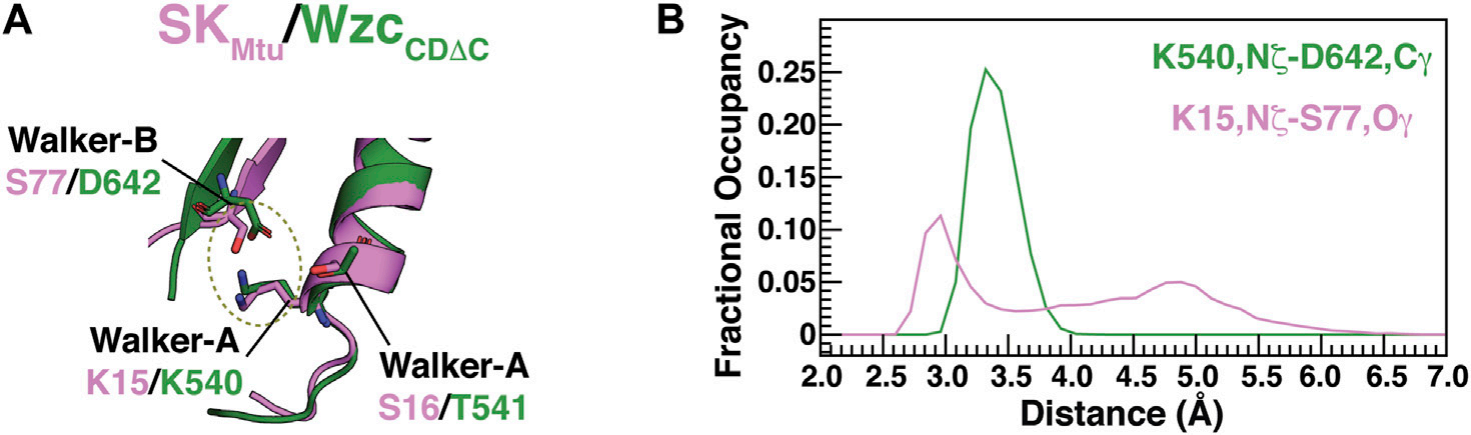
**(A)** Interactions that are characteristic of the open states of BY-kinases (OS) and SKs (WOS). S77 from the degraded Walker-B motif from SK_Mtu_ (pink) occupies approximately the same spatial position as the Walker-B D642 from Wzc_CDΔC_ (green) allowing the formation of a S77-K15 hydrogen bond reminiscent of the D642-K540 interaction in Wzc_CDΔC_. **(B)** Distribution of the K540,Nξ-D642,Cγ (green) and K15,Nξ-S77,Oγ (pink) distances from the REST2-generated ensembles of unliganded Wzc_CDΔC_ and SK_Mtu_, respectively.

As mentioned above, in a significant fraction of the structures that comprise the WOS of SK_Mtu_, the Walker-A K15 forms a hydrogen bond with the Walker-B S77 (Figure 1B), generating a conformation that mirrors that seen in the Wzc_CDΔC_ OS. In the latter case, the equivalent Walker-A Lys (K540) forms a salt bridge with the Walker-B D642. The β-strand housing the Walker-B motif in SK_Mtu_ is displaced relative to that in Wzc_CDΔC_, this enables S77 from the degraded Walker-B in the former to assume a spatial position that is approximately equivalent to the Walker-B D642 in the latter to facilitate an interaction with K15 (Figure 2A). It would therefore appear from our current results, that small local structural rearrangements allow the polar Ser to substitute for the now missing Walker-B Asp in preserving the interaction with the Walker-A K15 to maintain the open conformation (WOS) in SK_Mtu_. However, despite these similarities, there are differences in stability between the open conformations involving the Walker-A Lys of SKs and BY-kinases. In Wzc_CDΔC_, this interaction mode, as assessed through the K540,Nξ−D642,Cγ distance distribution, is well defined over the entire OS ensemble, showing a single peak with a maximum at ∼3.3 Å (green trace in Figure 2B). The corresponding distance, K15,Nξ–S77,Oγ in SK_Mtu_, shows a broad distribution with the dominant peak centered at ∼2.8 Å but containing only approximately 50% of the population in the WOS (pink trace in Figure 2B; this disorder is also evident from the bottom panel of Figure 1B). This suggests that while the orientation of the Walker-A Lys is well-defined in Wzc_CDΔC_, it is somewhat more dynamic in SK_Mtu_. The reasons for this difference are analyzed in detail below.

As mentioned earlier, the conformational dynamics of the LID and SB domains have been suggested to be critical for function in the SKs in serving to stabilize the reaction-compatible state, and in facilitating product release (Blanco et al., 2013). The dynamics of these entities have been suggested as suitable targets for drug discovery (Prado et al., 2016). For the sake of completeness, we also analyzed the local conformational variability in the REST2-generated ensembles of SK_Mtu_. There is, not unexpectedly, a slight decrease in overall flexibility upon binding ATP•Mg^2+^ (Supplementary Figure S5A), and transition from the WOS to the WCS. The LID domain that is the most dynamic region of the protein in the unliganded state remains so in the presence of ATP•Mg^2+^, though there is some decrease in its overall flexibility. The SB domain is also dynamic in both states of SK_Mtu_, albeit less so than the LID. To probe the presence of opening/closing motions of the SB domain with respect to the protein core, we defined a distance (d_47-109_, Supplementary Figure S5B, top left panel) between the Cα atoms of D47 (one of the more dynamic parts of the SB domain and therefore likely to sense these motions, if any) and R109 (near start of the LID domain; one of the more rigid parts of the protein in its ATP•Mg^2+^-bound state). In the absence of ATP•Mg^2+^ a somewhat bimodal distribution of distances is seen (Supplementary Figure S5B top right panel). Analyses of structures corresponding to the two maxima indicate an open conformation with greater separation between the LID and SB domains (Supplementary Figure S5B bottom right panel), and a more closed conformation where this distance is reduced (Supplementary Figure S5B bottom middle panel). Only the latter conformation is seen in the ATP•Mg^2+^-bound state (Supplementary Figure S4B bottom left panel).

### Conserved Features Define the Global Conformational States of P-Loop Enzymes

**FIGURE 3:**
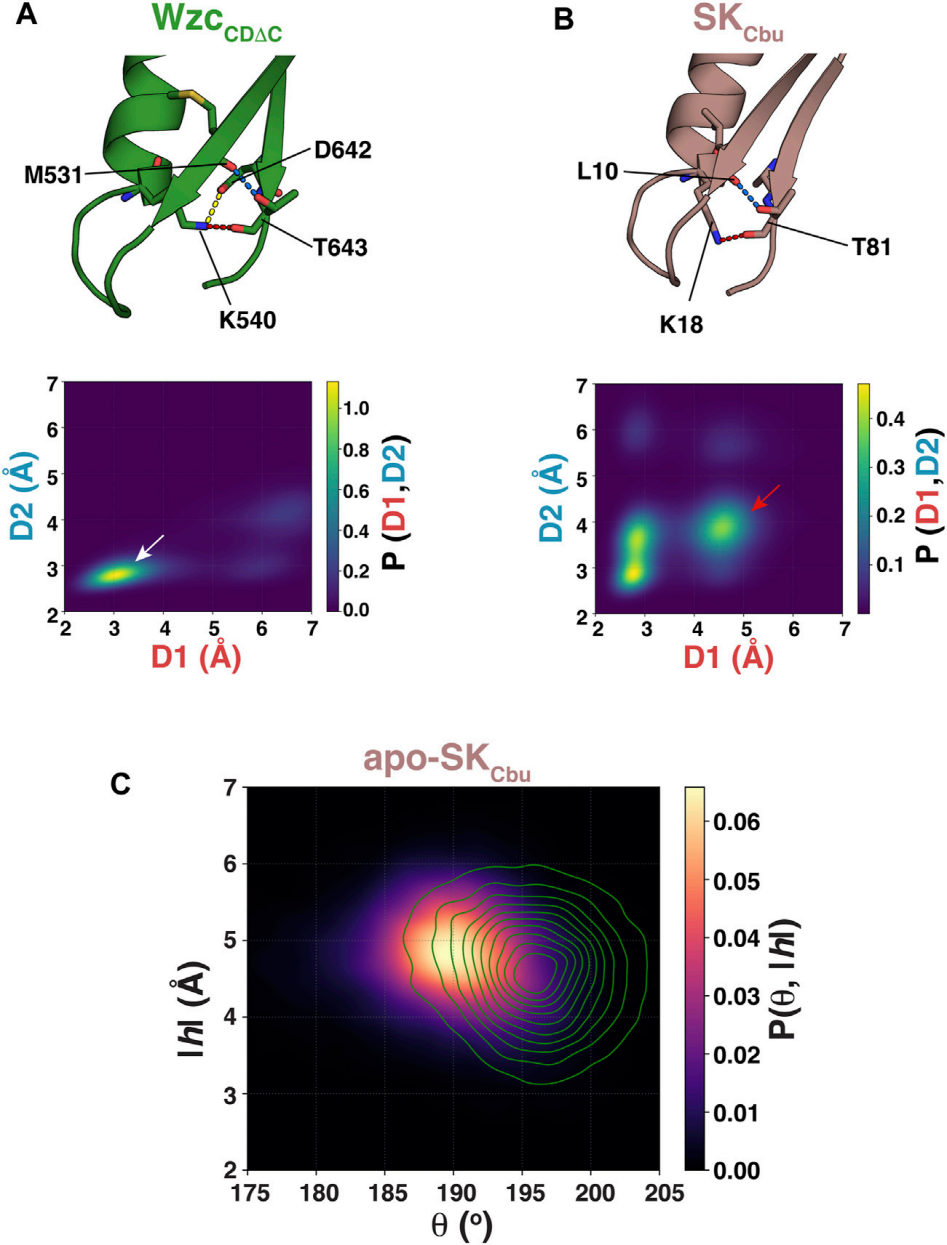
**(A)** Interactions of the Walker-A K540 with the Walker-B D642 (yellow dashed line), of K540 with T643 (red dashed line), and of T643 with M531 (blue dashed line) for Wzc_CDΔC_ in the OS are shown. **(B)** Interactions of the Walker-A K18 with Walker-B T81 (red dashed line) and of T81 with L10 (blue dashed line) for SK_Cbu_ in the WOS are shown. The lower panels, in each case, show the structural ensembles of unliganded Wzc_CDΔC_ and SK_Cbu_ projected onto the 2-dimensional space spanned by the {K540-T643 (D1: K540,Nξ−T643,O), T643-M531 (D2: T643,Oγ1−M531,O)} and {K18-T81 (D1: K18,Nξ−T81,O), T81-L10 (D2: T81,Oγ1−L10,O)} distances, respectively. **(C)** The structural ensemble of unliganded SK_Cbu_ is projected onto θ-|*h*| space and plotted using kernel density estimation. Also shown as green contours in the corresponding projection of the unliganded SK_Mtu_ ensemble.

While S77 of SK_Mtu_ appears to substitute for the Asp/Glu residue of a canonical Walker-B motif in maintaining the WOS through its interaction with the Walker-A Lys, this position is poorly conserved in SKs. In contrast to the ϕϕSLGGG sequence in SK_Mtu_, the consensus sequence for Walker-B motifs of SKs is ϕϕϕTGGG (Leipe et al., 2003); the corresponding consensus in BY-kinases is ϕϕϕϕDT (Leipe et al., 2002). Notably, in Wzc_CDΔC_, the conserved Walker-B Thr (T643) participates in multiple interactions that also serve to stabilize the OS (Figure 3A, top panel). T643 forms a hydrogen bond through its backbone carbonyl with the K540 sidechain (supplementing the interaction of the latter with D642), and an additional hydrogen bond through its sidechain hydroxyl with the backbone carbonyl of M531 on the adjacent β-sheet. Projection of the unliganded Wzc_CDΔC_ ensemble (Hajredini et al., 2020) onto the 2-dimensional space spanned by the K540-T643 (D1: K540,Nξ−T643,O) and T643-M531 (D2: T643,Oγ1−M531,O) distances, indicates a single major state (white arrow, Figure 3A, bottom panel). This robust network of interactions (K540-D642, K540-T643 and T643-M531) is the likely reason for the well-defined orientation of K540 in the OS (as is evident from the green trace in Figure 2B). This set of interactions is drastically altered in the CS (T643 now interacts T532, D642 interacts with T541, the latter interaction is necessary to optimally engage Mg^2+^) and this is the likely cause of the well-defined separation between in the OS and the CS upon comparing the apo- and ATP•Mg^2+^ complexes of Wzc_CDΔC_ (Supplementary Figures S3B,C).

To test whether a similar interaction mode involving the Walker-B Thr also stabilizes the WOS in SKs that carry the more conventional ϕϕϕTGGG sequence, we performed an additional set of REST2 simulations on the unliganded state of *Coxiella burnetii* SK (SK_Cbu_). Inspection of the structural ensemble of apo-SK_Cbu_ reveals the presence of hydrogen bonds between the backbone carbonyl and sidechain hydroxyl of the Walker-B Thr (T81) with the sidechain of the Walker-A K18 and the backbone carbonyl of L10 (that lies on the adjacent β-sheet), respectively (Figure 3B, top panel), mirroring the interaction mode seen in Wzc_CDΔC_. However, unlike in Wzc_CDΔC_, projection of the resultant ensemble onto the 2-dimensional space spanned by the K18-T81 (D1: K18,Nξ−T81,O) and T81-L10 (D2: T81,Oγ1−L10,Ο) distances reveals a more diffuse set of states (Figure 3B, bottom panel). Nevertheless, a disruption in the T81-K18 interaction leads to a corresponding disruption of the T81-L10 interaction (red arrow in Figure 3B, bottom panel) suggesting that these two sets of interactions are coupled as in Wzc_CDΔC_. It is of note that the canonical Walker-B Thr in SK_Cbu_ forms two sets of the hydrogen bonds involving both its backbone and its sidechain to stabilize the WOS. This contrasts a single hydrogen bond involving the sidechain of the unique Walker-B Ser (S77) in SK_Mtu_. One can therefore expect the WOS in SK_Cbu_ to be somewhat more closed than in SK_Mtu_. Inspection of Figure 3C shows that this is indeed the case with reduced θ values (SK_Cbu_: 190 ± 5°, SK_Mtu_: 196 ± 4°) and a corresponding increase in the |*h*| values (SK_Cbu_: 4.8 ± 0.6 Å, SK_Mtu_: 4.6 ± 0.7 Å).

**FIGURE 4:**
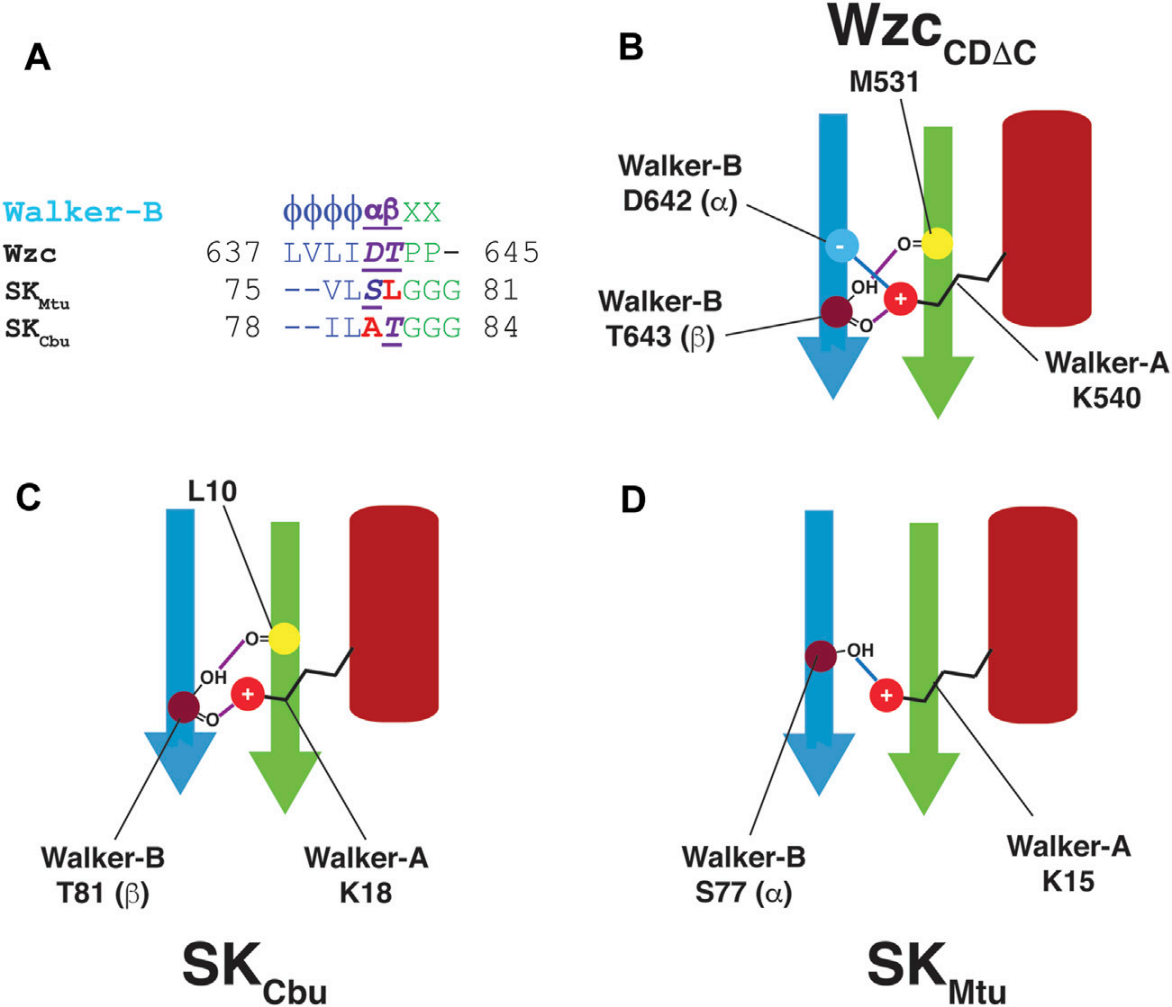
**(A)** Comparison of the Walker-B sequences in Wzc, SK_Mtu_ and SK_Cbu_. Wzc contains polar residues at both the α− and β−positions while only one of these positions is polar in SK_Cbu_ (β) and SK_Mtu_ (α) **(B)** The presence of polar residues at both the α− (D642) and β−positions (T643) in the Walker-B motif of Wzc_CDΔC_ allows the intricate network of interactions to appropriately align the Walker-A Lys (K540) leading to its well-defined orientation in the OS. These interactions are depicted schematically; hydrogen-bonds are indicated using purple and blue lines. Since only one of the α− or β−positions contain a polar residue in **(C)** SK_Cbu_ (β:T81) and **(D)** SK_Mtu_ (α:S77), only one of these sets of interactions (blue or purple) is preserved allowing a greater degree of orientational freedom in the WOS for the corresponding Walker-A Lys (K18 in SK_Cbu_, K15 in SK_Mtu_).

Based on the discussion above, an SK Walker-B motif may be generalized by the following sequence: ϕϕαβGGG; the α- and β-positions represent structural equivalents of the BY-kinase Walker-B Asp and Thr, respectively (Figure 4A). In unliganded Wzc_CDΔC_, both the α- and β-positions are optimal in that they contain polar residues. Thus, the Walker-A Lys (K540) can form hydrogen bonds with both the Walker-B Asp (D642 at the α−position) and with the Walker-B Thr (T643); the latter being a polar residue at the β−position is then able to utilize its sidechain to hydrogen bond with M531. This generates a mesh of interactions (shown schematically in Figure 4B) leading to a well-defined orientation of the Walker-A Lys (K540) in a stabilized OS. In contrast, the fact that only one of these two positions contains a polar residue for the two SKs analyzed here, with T81 (β) in SK_Cbu_ (Figure 4C) or S77 (α) in SK_Mtu_ (Figure 4D). Thus, only a subset of these interactions is possible in the SKs resulting in less well-defined orientation for the Walker-A Lys and a more diffuse WOS.

## Conclusion

Through enhanced sampling MD simulations on unliganded SK_Mtu_ and its ATP•Mg^2+^ complex, we have shown that as in the case of BY-kinases, the conformational landscapes of SKs contain open (WOS) and closed (WCS) states. The former is found in the unliganded state, while the latter is induced by the presence of nucleotide and Mg^2+^. In the WOS, the Walker-A K15 of SK_Mtu_ populates an extended conformation allowing the formation of a hydrogen bond with S77 of the degraded Walker-B motif that mimics the conserved Asp of the intact Walker-B motif in BY-kinases. In SK_Cbu_, that contains a Walker-B sequence that is more characteristic of P-loop kinases of the DxD group (Leipe et al., 2003), the conserved T81 forms a hydrogen-bond with the Walker-A K18 through its backbone while simultaneously using its polar sidechain to interact with the neighboring L10 backbone to stabilize the WOS. Thus, it appears that specific contacts between the Walker-A and Walker-B motifs, more specifically those involving the so-called catalytic Lys on the former, are necessary in P-loop-containing kinases to maintain the open conformation that cannot efficiently co-ordinate Mg^2+^ (and therefore, ATP•Mg^2+^) and is not conducive for chemistry. The importance of the hydrogen-bond involving the Walker-A Lys is evident from the fact that the absence of a Walker-B Asp as in the SKs, is compensated by the presence of a polar residue at a similar position. While the functional role the OS and CS and the interactions encoded within each state for the catalytic domain of Wzc has been extensively tested experimentally (Hajredini et al., 2020; Hajredini and Ghose, 2021), we expect that the computational analyses presented here will provide guidelines for similar experimental validation of key functional features in SKs in particular, and in P-loop kinases, in general.

## Data Availability

The raw data supporting the conclusions of this article will be made available by the authors, without undue reservation.
