# Supplementary material for: A Conserved Structural Role for the Walker-A Lysine in P-Loop Containing Kinases
Source: Front Mol Biosci. 2021 Oct 1;8:747206. doi: 10.3389/fmolb.2021.747206 (PMC8517177; doi:10.3389/fmolb.2021.747206)
Supplement: Supplementary file 1 [file DataSheet1.PDF]

**Supplementary Material**

for

# A conserved structural role for the Walker-A lysine in P-loop containing kinases

Fatlum Hajredini and Ranajeet Ghose

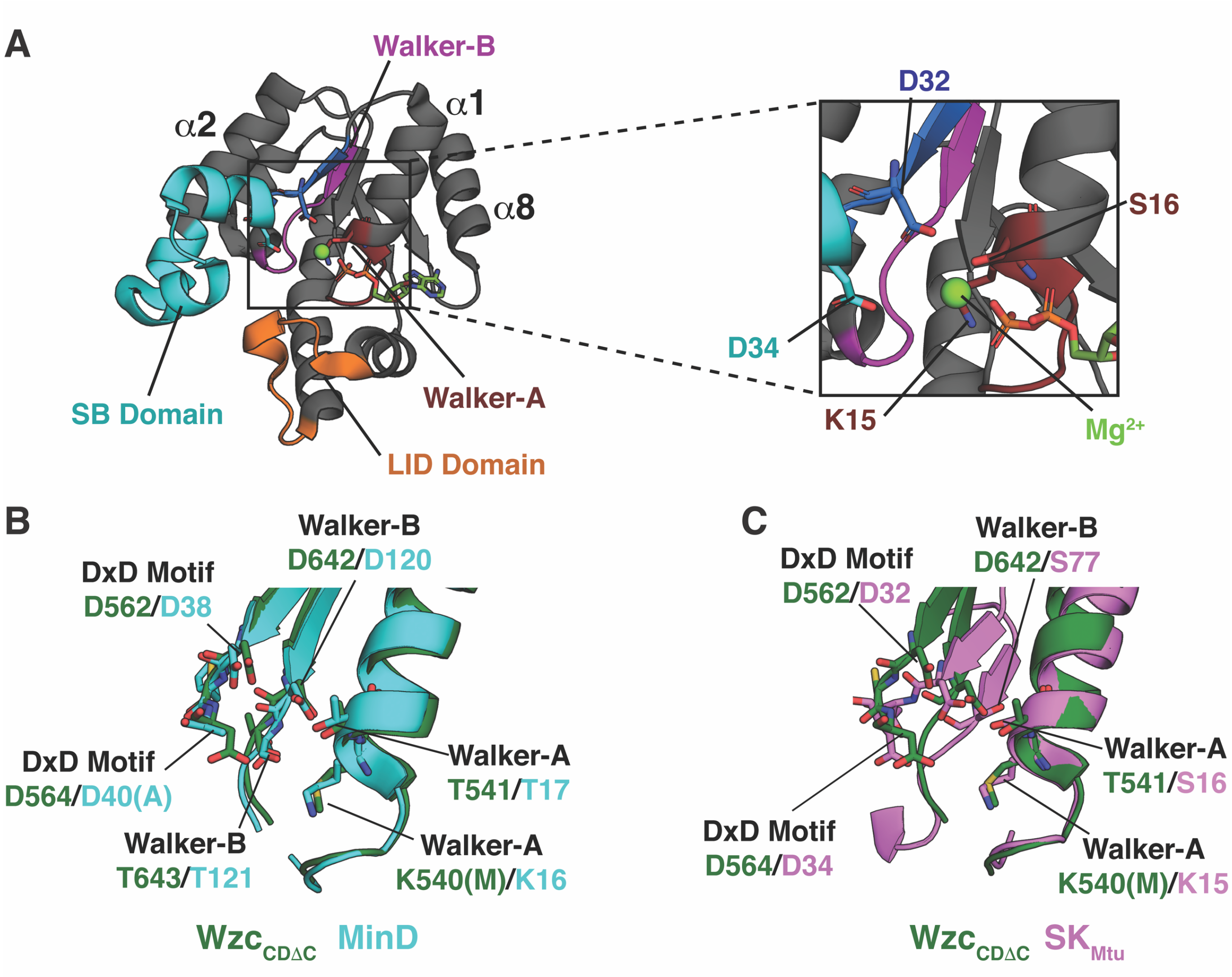


**Figure S1**. **(A)** Structure of *Mycobacterium tuberculosis* shikimate kinase ($SK_{Mtu}$). The shikimate binding (SB) and LID domains are colored cyan and orange, respectively. The Walker-A, Walker-B and the DxD motifs are colored brown, purple and dark blue, respectively. An expanded view of the catalytic site is shown on the right panel; key residues of the Walker-A (K15, S16) and DxD (D32, D34) motifs are indicated. Comparison of the catalytic site geometry of $Wzc_{CD\Delta C}$ (K540M mutant, green) with **(B)** MinD (D40A mutant, cyan) and **(C)** $SK_{Mtu}$ (purple); corresponding residues are indicated in each case. Note that like $Wzc_{CD\Delta C}$, MinD also contains both a Walker-B Asp and a DxD motif.

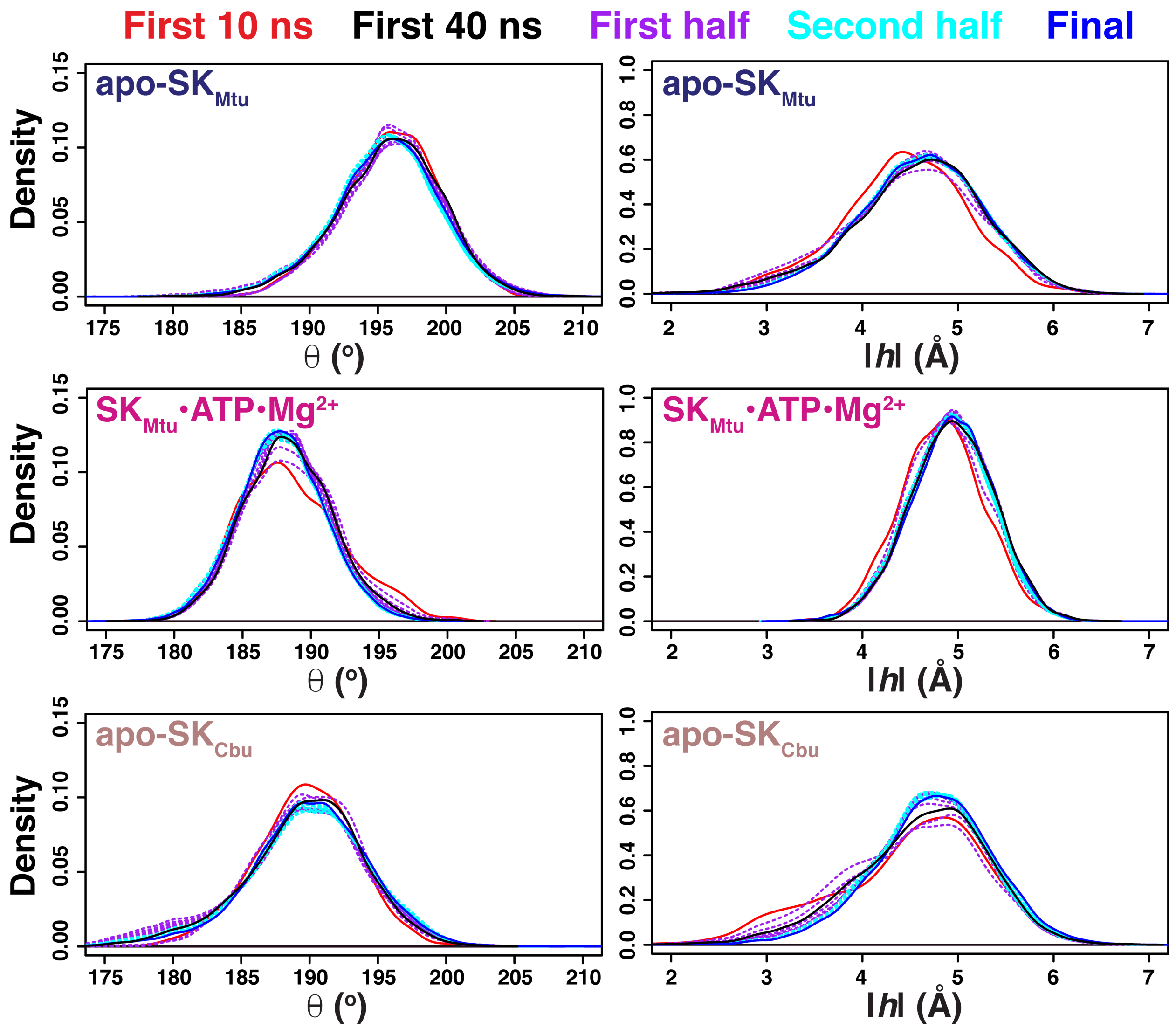


**Figure S2.** Convergence of the θ and |*h*| distributions in the REST2 simulations on apo-$SK_{Mtu}$ (top panels), $SK_{Mtu}$•ATP•$Mg^{2+}$ (middle panels) and apo-$SK_{Cbu}$ (bottom panels). Cumulative densities of states along the θ (left panels) and |*h*| (right panels) dimensions calculated in 10 ns increments using kernel density estimation are shown as colored lines. Data for various durations are shown together with the final distributions (blue solid line) obtained over the last 160 ns. As is evident, the distributions obtained after 40 ns (black solid line) are essentially the same as the final distributions in contrast to the large deviations of the distributions calculated from the first 10 ns (red solid line) of the 200 ns trajectories. Also shown for reference are distributions calculated over 10 ns intervals from the first half (purple dashed lines) and second half (cyan dashed lines) of the simulations.

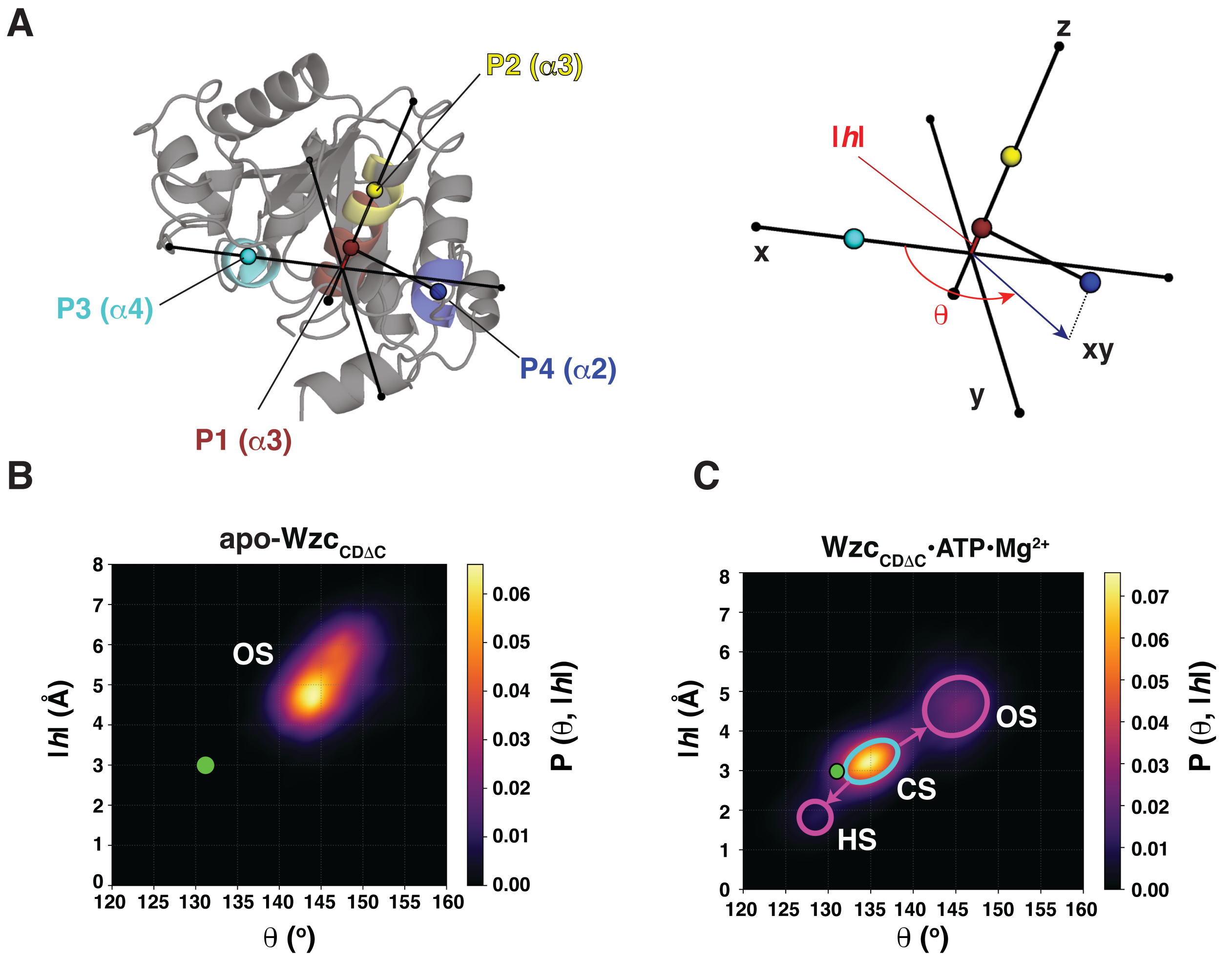


**Figure S3. (A)** Points P1 (brown), P2 (yellow), P3 (cyan) and P4 (blue) that define the cylindrical coordinate frame used to analyze the REST2-generated structural ensembles of Wzc$_{CD\Delta C}$ are indicated on its structure. The definitions of the angle θ and the rise |*h*| are shown on the right panel. The REST2-generated ensembles for apo-Wzc$_{CD\Delta C}$ **(B)** and the Wzc$_{CD\Delta C}$•ATP•$Mg^{2+}$ complex **(C)** are shown projected onto θ-|*h*| space. Only an open state (OS) is populated in unliganded Wzc$_{CD\Delta C}$. The presence of ATP•$Mg^{2+}$ leads to the partial occupancy of a dominant closed state (CS) and a sparsely populated hyper-closed state (HS). The green filled circle represents the conformation seen in the crystallographic monomers (PDB: 3LA6).

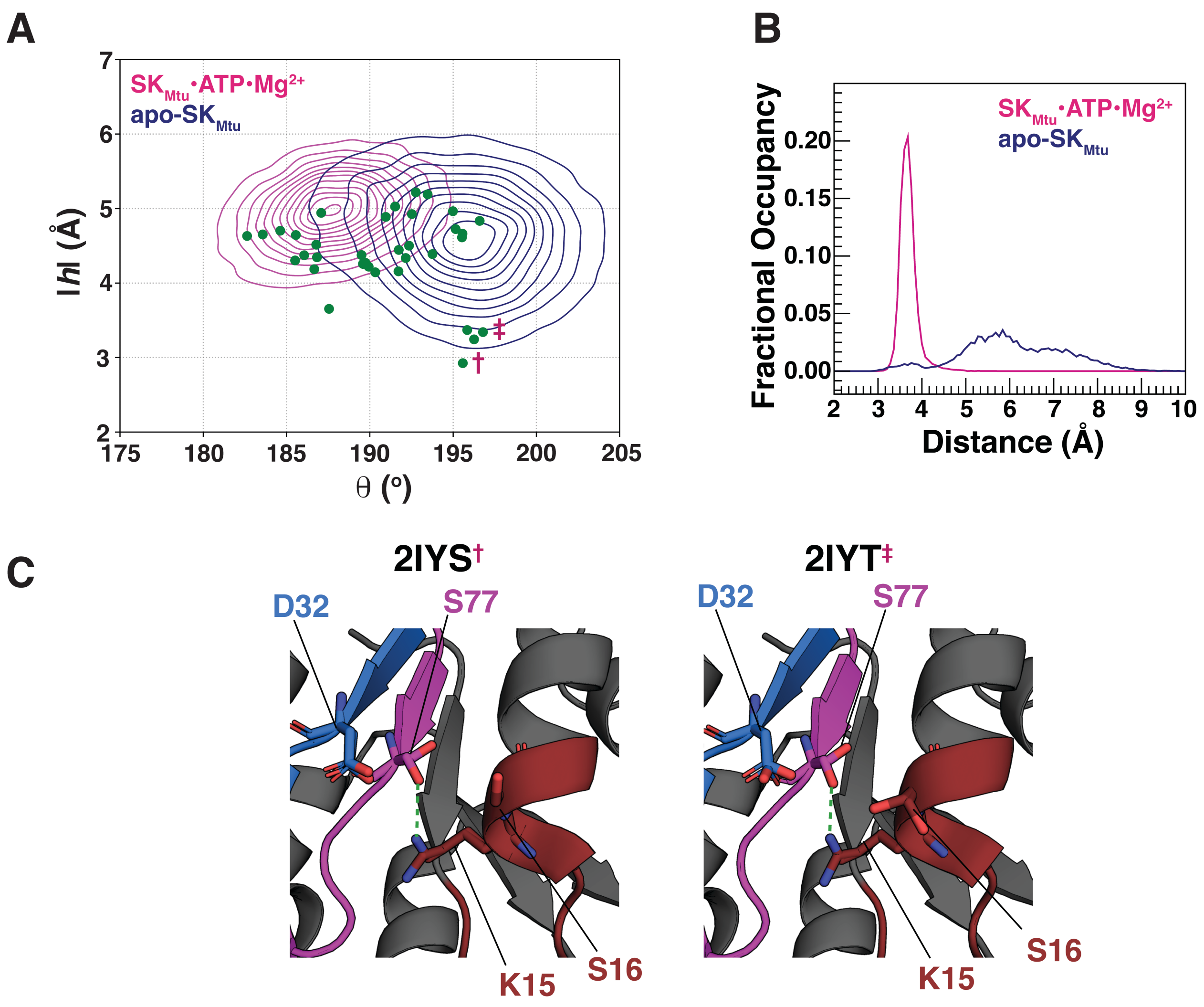


**Figure S4.** **(A)** The conformations sampled in the REST2-generated ensembles of apo-SK$_{Mtu}$ (dark blue) and the SK$_{Mtu}$•ATP•$Mg^{2+}$ complex (pink) are plotted using kernel density estimation and represented as contours. Projections of the conformations seen in the crystal structures of several SK enzymes (and their complexes) are indicated by the green circles. The '†' and '‡' indicate two sets of $Mg^{2+}$-free structures that are the most open with the highest θ and the lowest |*h*| values. **(B)** Distributions of the distance between the D32, Cγ (DxD motif) and the S16, Oγ (Walker-B motif) atoms for apo-SK$_{Mtu}$ (dark blue) and the SK$_{Mtu}$•ATP•$Mg^{2+}$ complex (pink) in their respective REST2-generated ensembles are shown. **(C)** Crystal structures of unliganded SK$_{Mtu}$, indicated by the '†' and '‡' symbols in **(A)**, illustrating proximity (green dashed line) between the catalytic Walker-A Lys (K15) and the Walker-B S77.

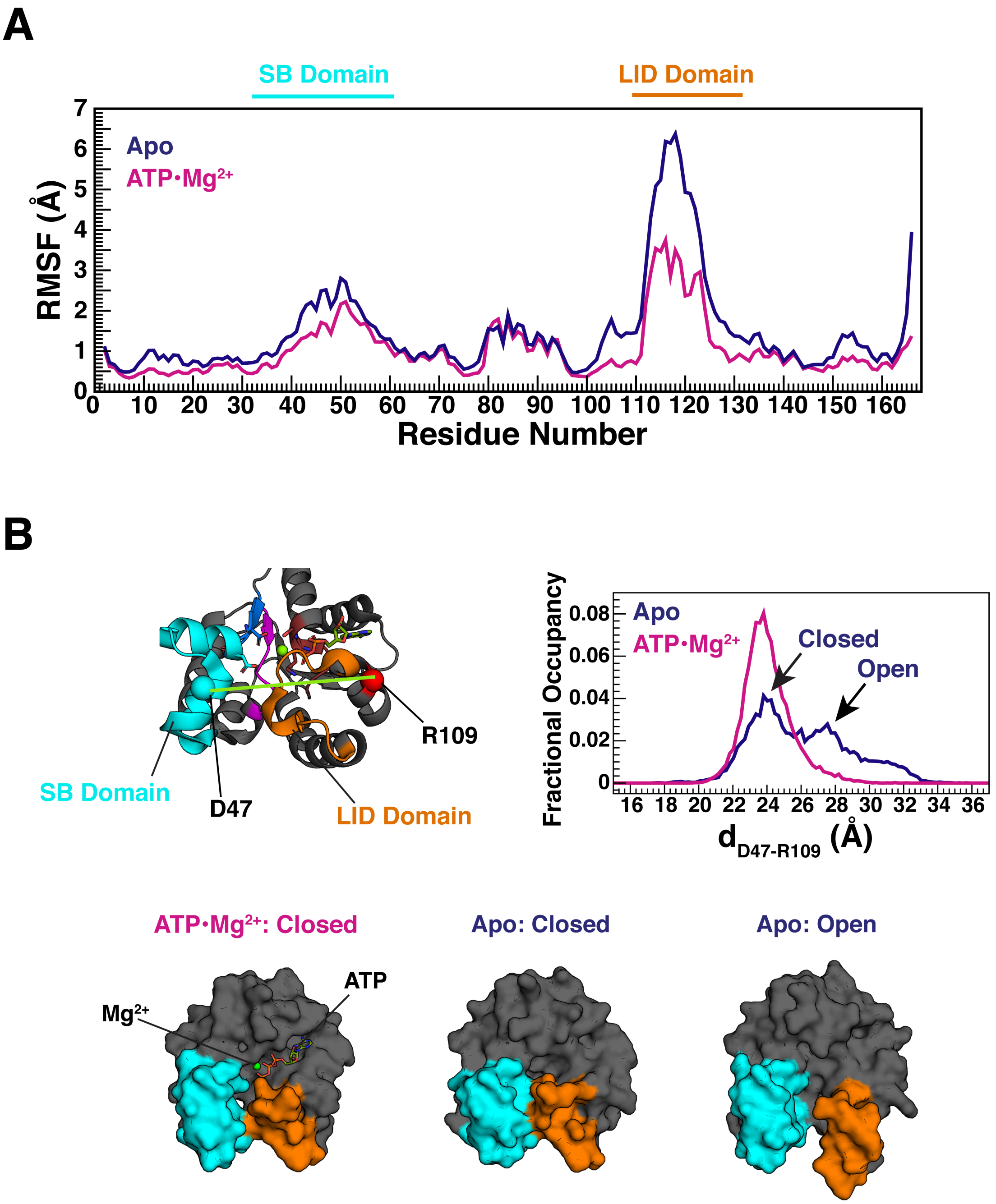


**Figure S5. (A)** Root mean squared fluctuations (RMSF) for apo-$SK_{Mtu}$ (dark blue) and the $SK_{Mtu}$•ATP•$Mg^{2+}$ complex (pink) in their respective REST2-generated ensembles are plotted against residue number. The positions of the SB (cyan) and LID (orange) domains along the sequence are indicated. **(B)** Distribution of the distance ($d_{47\text{-}109}$, shown as a light green line on the left panel) between the Cα atoms of D47 (SB domain) and R109 (start of the LID domain) in the REST2-generated ensembles of apo-$SK_{Mtu}$ (dark blue) and the $SK_{Mtu}$•ATP•$Mg^{2+}$ complex (pink), are shown. The bottom panel shows representative structures drawn from the maxima of the distributions in each case.

**Table S1. Shikimate kinase crystal structures used in the analysis**

| PDB ID | Organism | Ligand |
|---|---|---|
| 1L4U | *M. tuberculosis* | ADP•$Mg^{2+}$ |
| 1L4Y | *M. tuberculosis* | ADP•$Mg^{2+}$ |
| 1U8A | *M. tuberculosis* | ADP•SA |
| 1WE2 | *M. tuberculosis* | ADP•$Mg^{2+}$•SA |
| 1ZYU | *M. tuberculosis* | AMPPCP•SA |
| 2DFN | *M. tuberculosis* | ADP•SA |
| 2DFT | *M. tuberculosis* | ADP•$Mg^{2+}$ |
| 2G1J | *M. tuberculosis* | Apo |
| 2IYQ | *M. tuberculosis* | ADP•SA |
| 2IYR | *M. tuberculosis* | SA |
| 2IYS | *M. tuberculosis* | SA |
| 2IYT | *M. tuberculosis* | Apo |
| 2IYU | *M. tuberculosis* | ADP |
| 2IYV | *M. tuberculosis* | ADP |
| 2IYW | *M. tuberculosis* | ATP•$Mg^{2+}$ |
| 2IYX | *M. tuberculosis* | Apo |
| 2IYY | *M. tuberculosis* | pSA |
| 2IYZ | *M. tuberculosis* | ADP•pSA |
| 3BAF | *M. tuberculosis* | AMPPNP•SA |
| 4BQS | *M. tuberculosis* | ADP•SA |
| 2G1K | *M. tuberculosis* | SA |
| 1ZUH | *H. Pylori* | Apo |
| 1ZUI | *H. pylori* | SA•$PO_4^{2-}$ |
| 3HR7 | *H. pylori* | Apo |
| 3MRS | *H. pylori* | Apo |
| 3MUF | *H. pylori* | ADP•pSA |
| 1E6C | *E. chrysanthemi* | Apo (Walker-A (K15M) |
| 1SHK | *E. chrysanthemi* | Apo |
| 2SHK | *E. chrysanthemi* | ADP•$Mg^{2+}$ |
| 3TRF | *C. burnetii* | Apo |
| 3VAA | *B. thetaiotaomicron* | Apo |
| 4Y0A | *A. baumannii* | SA |
| 2PT5 | *A.aeolicus* | Apo |
| 1VIA | *C. jejuni* | Apo |
| 1KAG | *E. coli* | Apo |

The liganded states and mutations (if any are indicated; SA shikimate). Structures are labeled as "apo" if they do not contain nucleotides (or variants thereof) and/or shikimate. The structure of *H. pylori* SK (3N2E) complexed to an inhibitor has not been included in the analysis.

**Table S2. Residues used for points P1-P4 to define the cylindrical coordinate frame in SKs from a variety of organisms**

| Organism | P1 | P2 | P3 | P4 |
|---|---|---|---|---|
| *M. tuberculosis* | 15-19 | 21-25 | 33-37 | 155-159 |
| *H. pylori* | 14-18 | 20-24 | 32-36 | 150-154 |
| *D. chrysanthemi* | 15-19 | 21-25 | 33-37 | 157-161 |
| *C. burnetii* | 18-22 | 24-28 | 36-40 | 161-165 |
| *B. thetaiotaomicron* | 14-18 | 20-24 | 32-36 | 162-166 |
| *A. baumannii* | 31-35 | 37-41 | 49-53 | 172-176 |
| *A. aeolicus* | 13-17 | 19-23 | 31-35 | 150-154 |
| *C. jejuni* | 17-21 | 23-27 | 35-39 | 153-157 |
| *E. coli* | 17-21 | 23-27 | 35-39 | 159-163 |
